# Adaptive Brain Shut-Down Counteracts Neuroinflammation in the Near-Term Ovine Fetus

**DOI:** 10.3389/fneur.2014.00110

**Published:** 2014-06-30

**Authors:** Alex Xu, Lucien Daniel Durosier, Michael G. Ross, Robert Hammond, Bryan S. Richardson, Martin G. Frasch

**Affiliations:** ^1^Department of Obstetrics and Gynecology, Western University, London, ON, Canada; ^2^Department of Obstetrics and Gynaecology and Department of Neurosciences, CHU Sainte-Justine Centre de Recherche, Université de Montréal, Montréal, QC, Canada; ^3^Department of Obstetrics and Gynecology, LA BioMed at Harbor-UCLA Medical Center, Torrance, CA, USA; ^4^Department of Pathology, Western University, London, ON, Canada

**Keywords:** fetus, microglia, ECoG, EEG, hypoxia, acidemia, labor, sheep

## Abstract

**Objective:** Repetitive umbilical cord occlusions (UCOs) in ovine fetus leading to severe acidemia result in adaptive shut-down of electrocortical activity [electrocorticogram (ECoG)] as well as systemic and brain inflammation. We hypothesized that the fetuses with earlier ECoG shut-down as a neuroprotective mechanism in response to repetitive UCOs will show less brain inflammation and, moreover, that chronic hypoxia will impact this relationship.

**Methods:** Near-term fetal sheep were chronically instrumented with ECoG leads, vascular catheters, and a cord occluder and then underwent repetitive UCOs for up to 4 h or until fetal arterial pH was <7.00. Eight animals, hypoxic prior to the UCOs (SaO_2_ <55%), were allowed to recover 24 h post insult, while 14 animals, 5 of whom also were chronically hypoxic, were allowed to recover 48 h post insult, after which brains were perfusion-fixed. Time of ECoG shut-down and corresponding pH were noted, as well as time to then reach pH <7.00 (ΔT). Microglia (MG) were counted as a measure of inflammation in gray matter layers 4–6 (GM4–6) where most ECoG activity is generated. Results are reported as mean ± SEM for *p* < 0.05.

**Results:** Repetitive UCOs resulted in worsening acidosis over 3–4 h with arterial pH decreasing to 6.97 ± 0.02 all UCO groups’ animals, recovering to baseline by 24 h. ECoG shut-down occurred 52 ± 7 min before reaching pH <7.00 at pH 7.23 ± 0.02 across the animal groups. MG counts were inversely correlated to Δ*T* in 24 h recovery animals (*R* = −0.84), as expected. This was not the case in normoxic 48 h recovery animals, and, surprisingly, in hypoxic 48 h recovery animals, this relationship was reversed (*R* = 0.90).

**Conclusion:** Adaptive brain shut-down during labor-like worsening acidemia counteracts neuroinflammation in a hypoxia- and time-dependent manner.

## Introduction

Human clinical studies with umbilical cord blood gas and pH assessment at birth indicate an increasing risk for neonatal adverse outcome and longer-term sequellae including cerebral palsy with pH values <7.00 (1–3). Additionally, growth-restricted infants with chronic hypoxemia due to placental dysfunction are at greater risk for concerning acidemia at birth and thereby subsequent adverse neurological outcomes due to superimposed acute hypoxemia during labor (4–7). This is supported by studies in the ovine fetus showing that pre-existing hypoxia alters cerebral and cardiovascular responses to labor-like umbilical cord occlusions (UCOs) (8). This has led to the use of electronic fetal heart rate (FHR) monitoring as the main stay for the assessment of fetal health during labor (1–3). The absence of FHR decelerations along with presence of FHR variability is highly predictive for normal fetal blood gas/pH at birth (1–3). However, clinical FHR monitoring has a low positive predictive value for concerning acidemia at birth (~50%), so there is continued need for improving existing technologies for the detection of fetal hypoxic-acidemia during labor. (1–3)

We recently studied patterns of electrocortical activity [electrocorticogram (ECoG)] and FHR in the near-term ovine fetus in response to repetitive UCOs insults as might be seen in human labor, to delineate the time-course and correlation of ECoG change with worsening acidemia (7). There were consistent changes in ECoG with amplitude suppression and frequency increase during FHR decelerations in association with pathological decreases in fetal arterial blood pressure (ABP). These changes in ECoG suggested an “adaptive brain shut-down” and occurred on average 50 min prior to attaining a severe degree of acidemia with fetal arterial pH <7.00. Translational implications are that fetal ECoG monitoring can improve the positive predictive value of FHR monitoring for worsening acidemia at birth. We have shown that emergence of pathological decreases in ABP in response to UCO-triggered FHR decelerations is not required for the observed correlated ECoG–FHR changes. This suggests that these changes are induced neurally in the context of an adaptive brain shut-down (7). The relationship between these cerebral responses and acidemia-triggered brain inflammation remains unknown (7, 9). If neuroinflammation is diminished by adaptive brain shut-down, then expedited delivery during labor when observing this “ECoG–FHR shut-down pattern” may improve postnatal brain development by reducing the risk for sustained postnatal neuroinflammation.

Based on our previous findings for fetal ECoG (7, 10), we hypothesized that fetuses with an earlier adaptive brain shut-down will show less neuroinflammation due to the neuroprotective effect of decreasing cerebral metabolic rate. Consequently, in the present study we quantified neuroinflammation in response to worsening acidemia as microglial activation in the fetal brain at 24 and 48 h following the cessation of the UCOs.

## Materials and Methods

### Surgical preparation

Twenty two near-term ovine fetuses [124 ± 1 days gestational age (GA), term = 145 days] of mixed breed were surgically instrumented. The anesthetic and surgical procedures and post-operative care of the animals have been previously described (4, 5). Briefly, polyvinyl catheters were placed in the right and left brachiocephalic arteries and the right cephalic vein. Stainless steel electrodes were sewn onto the fetal chest to monitor the electrocardiogram (EKG). Stainless steel electrodes were additionally implanted biparietally on the dura for the recording of ECoG. An inflatable silicon rubber cuff (*In vivo* Metric, Healdsburg, CA, USA) for UCO induction was also placed around the proximal portion of the umbilical cord and secured to the abdominal skin. Once the fetus was returned to the uterus, a catheter was placed in the amniotic fluid cavity and another in the maternal femoral vein. Antibiotics were administered intravenously to the mother (0.2 g trimethoprim and 1.2 g sulfadoxine, Schering Canada Inc., Pointe-Claire, QC, Canada) and the fetus and into the amniotic cavity (1 million IU penicillin G sodium, Pharmaceutical Partners of Canada, Richmond Hill, ON, Canada). Amniotic fluid lost during surgery was replaced with warm saline. The uterus and abdominal wall incisions were sutured in layers and the catheters exteriorized through the maternal flank and secured to the back of the ewe in a plastic pouch.

Postoperatively, animals were allowed 4 days to recover prior to experimentation and daily antibiotic administration was continued. Arterial blood was sampled for evaluation of fetal condition and catheters were flushed with heparinized saline to maintain patency. Animals were 128 ± 1 days GA on the first day of experimental study. Animal care followed the guidelines of the Canadian Council on Animal Care and was approved by the University of Western Ontario Council on Animal Care.

### Experimental procedure

The animals were studied over a ~6 h period in three groups. Fetal chronic hypoxia was defined as arterial O_2_Sat <55% as measured on post-operative days 1–3 and at baseline prior to beginning the UCOs. The first group comprised eight animals spontaneously hypoxic that were subjected to repetitive UCOs followed by 24 h recovery with subsequent necropsy to assess neuroinflammation (H/UCO 24). While these ECoG and neuroinflammation results have been published (7, 11), they were not interpreted together and are included here to address the new question of the relationship between the degree of adaptive brain shut-down and neuroinflammation. The second group comprised five fetuses that were also spontaneously hypoxic but studied at 48 h recovery (H/UCO 48). The third group of fetuses was normoxic (O_2_Sat >55% before UCOs) and formed an N/UCO 48 h recovery group (*n* = 9, N/UCO 48). That is, the second and third UCO groups were allowed to recover for 48 h after the UCOs with subsequent necropsy to assess neuroinflammation.

The protocol for the first group has been reported (7, 11) and is essentially similar in the objective to the protocol described below in that it mimics uterine contractions during human labor. The protocol of the H/UCO 48 and N/UCO 48 groups has also been reported (12, 13). The difference between the groups with regard to mimicking the uterine contractions during labor is that in the H/UCO group with 24 h recovery time, the UCOs’ frequency was increased with each UCO being a complete one, while in the H/UCO and N/UCO groups with 48 h recovery the frequency was kept constant and the completeness of the UCO series was increased progressively. The end result was the same: a cumulative, worsening acidemia. Hence, we believe that this difference in experimental design has no confounding impact on our findings. After a 1–2 h baseline control period, the second and third groups of animals underwent mild, moderate, and severe series of repetitive UCOs by graduated inflation of the occluder cuff with a saline solution. During the first hour following the baseline period, mild variable FHR decelerations were performed with a partial UCO for 1 min duration every 2.5 min, with the goal of decreasing FHR by ~30 bpm, corresponding to an ~50% reduction in umbilical blood flow (14, 15). During the second hour, moderate variable FHR decelerations were performed with increased partial UCO for 1 min duration every 2.5 min with the goal of decreasing FHR by ~60 bpm, corresponding to an ~75% reduction in umbilical blood flow (15). Animals then underwent severe variable FHR decelerations with complete UCO for 1 min duration every 2.5 min until the targeted fetal arterial pH of <7.0 was detected or 2 h of severe UCO had been carried out, at which point the repetitive UCOs were terminated. These animals were then allowed to recover for 48 h following the last UCO. Fetal arterial blood samples were drawn at baseline, at the end of the first UCO of each series (mild, moderate, severe), and at 20 min intervals (between UCOs) throughout each of the series, as well as at 1, 24, and 48 h of recovery. For each UCO series, blood gas sample and the 24 h recovery sample, 0.7 mL of fetal blood was withdrawn, while 4 mL of fetal blood was withdrawn at baseline, at pH nadir <7.00, and at 1 and 48 h of recovery. The amounts of blood withdrawn were documented for each fetus and replaced with an equivalent volume of maternal blood at the end of day 1 of study.

All blood samples were analyzed for blood gas values, pH, glucose, and lactate with an ABL-725 blood gas analyzer (Radiometer Medical, Copenhagen, Denmark) with temperature corrected to 39.0°C. Plasma from the 4 mL blood samples was frozen and stored for cytokine analysis, and will be reported separately.

After the 24 or 48 h recovery blood sample, the ewe and the fetus were killed by an overdose of barbiturate (30 mg sodium pentobarbital IV, MTC Pharmaceuticals, Cambridge, ON, Canada). A post mortem was carried out during which fetal sex and weight was determined and the location and function of the umbilical occluder were confirmed. The fetal brain was perfusion-fixed and subsequently dissected and processed for later immunohistochemical study as previously reported (11).

### Data acquisition and analysis

**Figure 1:**
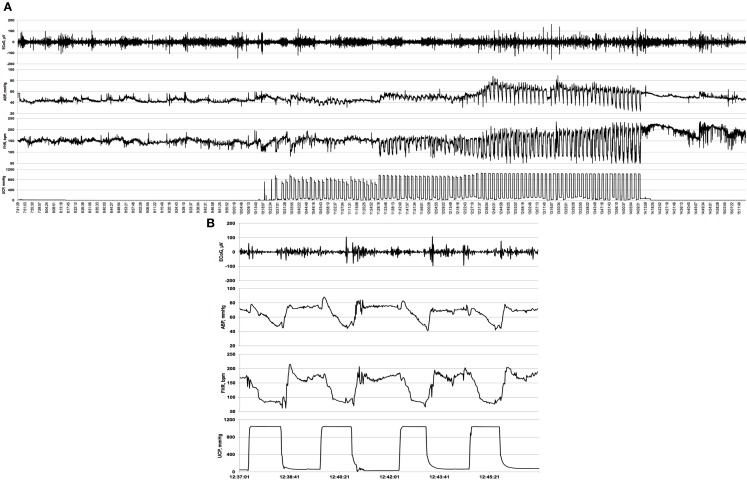
**Example of an individual electrocorticogram (ECoG) response to repetitive umbilical cord occlusions (UCOs)**. **(A)** A complete 5 h recording is shown with baseline, mild, moderate, and severe UCOs. UCOs occurrences are indicated by the UCP channel: umbilical contraction pressure increases correspond to an occlusion of the umbilical cord. Note emergence of the adaptive brain shut-down pattern visible in ECoG- and fetal heart rate (FHR) synchronization and the accompanying changes in arterial blood pressure (ABP). **(B)** Ten minutes zoomed-in window of this synchronized ECoG–FHR pattern.

A computerized data acquisition system was used to record fetal arterial and amniotic pressures, the ECG and ECoG electrical signals, as previously described (16), which were monitored continuously throughout the baseline, UCO series, and first hour of the recovery period (Figure 1). Arterial and amniotic pressures were measured using Statham pressure transducers (P23 ID; Gould Inc., Oxnard, CA, USA). ABP was determined as the difference between instantaneous values of arterial and amniotic pressures. A PowerLab system was used for data acquisition and analysis (Chart 7 For Windows, ADInstruments Pty Ltd., Castle Hill, NSW, Australia).

Pressures, ECG, and ECoG were recorded and digitized at 1000 Hz for further study. For ECG, a 60 Hz notch filter was applied, while for ECoG, a band pass 0.3–30 Hz filter was used. FHR was triggered and calculated online from arterial pressure systolic peaks.

Averaged values of FHR and ABP were calculated from artifact-free recordings of 1 h of baseline, as well as between and during each consecutive variable FHR deceleration induced by the mild, moderate, and severe UCOs as previously reported (12).

Electrocorticogram was sampled down to 100 Hz prior to the analysis. For each animal, mean values of the ECoG amplitudes were determined at baseline as well as during and between UCO for each of the UCO deceleration series.

#### Immunohistochemistry

The presence of microglia (MG) in brain tissue sections was determined by avidin–biotin-peroxidase complex enhanced immunohistochemistry (Vectastain Elite; Vector Laboratories, Burlingame, CA, USA) as previously reported (9, 11). To reduce staining variability, all immunohistochemistry was performed on the same day with the same batch of antibody and solutions. Tissue sections were incubated with an anti-IBA1 rabbit polyclonal antibody (1:500, Wako Industries, Richmond, VA, USA), a robust marker for sheep MG, with detection of bound antibody obtained following incubation in Cardassian DAB Chromogen (Biocare Medical, Concord, CA, USA).

Brain regions that were selected from each animal for analysis were taken from a coronal section of blocked cerebral hemisphere tissue at the level of the mammillary bodies and included the parasagittal and convexity cerebral gray matter, periventricular white matter, thalamus, CA1, dentate gyrus (DG), and the combined CA2 and CA3 regions of the hippocampus. Each of the gray matter regions was further divided into sub-regions combining layers 1, 2, and 3 and layers 4, 5, and 6. Image analysis was performed with a transmitted light microscope (Leica DMRB, Leica-Microsystems, Wetzler, Germany) at 40× magnification. Positive MG cell immunostaining was quantified with an image analysis program (Image Pro Plus 6.0, Media Cybernetics, Silver Spring, MD, USA). The image analysis system was first calibrated for the magnification settings that were used, and thresholds were established to provide even lighting and no background signal. Six high-power field (HPF) photomicrographs (HPF area = 7 cm^2^) per brain region/subregion per animal were collected as a 24 bit RGB color modeled image. The same illumination setting was applied to all images for all of the brain regions. Using the Image Pro Plus’ RGB color range selection tool, color samplings of positive DAB stained areas were obtained from multiple brain regions and tested for specificity against the negative control. Appropriate ranges of color were selected showing positive contiguous cytoplasmic staining as a criterion for MG cell count scoring, which were then applied uniformly to calibrated images for all brain regions. Scoring was performed in a blinded fashion to the N/UCO, H/UCO, and control (no UCO) groups. Specifically, to perform the MG counts normalization procedure for inter-group comparisons outlined below, the respective control groups were analyzed as follows: a hypoxic control group (i.e., no UCOs, same recovery time, *N* = 5) matched to H/UCO 24, another normoxic control group matched to N/UCO 48 (no UCOs, same recovery time, *N* = 10), and a hypoxic control group (i.e., no UCOs, *N* = 4). In the current study, our focus is on the neuroinflammation in the gray matter layers 4–6 (GM4–6) where most ECoG activity is generated. Neuroinflammation results in other brain regions for H/UCO 24 have been reported (11) and for H/UCO 48 and N/UCO 48 will be reported separately.

#### Normalization of MG counts to allow comparison between the 24 and 48 h recovery groups

Different observers scored H/UCO 24 and H/UCO 48 and N/UCO 48 groups. This is due to the fact that the respective research projects took place at different time points. This necessitated a normalization of each group’s MG counts to eliminate any observer-dependent variability in MG scoring thus allowing an inter-group comparison of MG counts. To achieve that goal, we calculated average values of MG counts in each brain region for the control animals in each of the three groups. The values were then used to normalize the individual regional MG counts in each of the intervention groups, thus allowing an inter-group comparison.

#### Analysis of the relation between neuroinflammation and ECoG activity

Data from the first group of animals (7, 11) were pooled together with the current findings in the second and third groups. As noted, in the first group, eight animals, all hypoxic prior to the UCOs, were allowed to recover 24 h post insult, while the 14 animals currently studied formed the cohort that was allowed to recover 48 h post insult, after which brains were perfusion-fixed. The individual time of fetal adaptive brain shut-down onset was noted as observed at the start of the synchronized ECoG/FHR changes. The difference between this time and the time to then reach the target pH <7.00 was calculated as Δ*T*. The normalized MG counts in the GM4–6 were then correlated to Δ*T*.

### Statistical analysis

Normal data distribution was tested using Kolmogorov–Smirnov test followed by parametric or non-parametric tests, as appropriate. Arterial pH, lactate, and base excess (BE) measurements in response to repetitive UCOs at the onset the adaptive brain shut-down and at pH nadir were compared with the corresponding baseline values by one-way repeated-measures analysis of variance (ANOVA) with Student–Newman–Keuls *post hoc* analysis. One-way repeated-measures ANOVA followed by Holm–Sidak (versus baseline) or Student–Newman–Keuls tests for multiple comparisons have been used to assess differences in ECoG and cardiovascular responses to UCOs within each UCO group. Differences in ECoG and cardiovascular alterations during the synchronized ECoG–FHR pattern were tested using *t*-test or signed rank test (i.e., pairwise during versus between UCO during adaptive brain shut-down).

Between group differences of the values of the fetal body and brain weights as well as brain/body ratios were assessed using ANOVA or Kruskal–Wallis ANOVA on ranks with Holm–Sidak or Dunn’s tests for multiple pairwise comparisons, respectively. Between group differences of the values of the arterial O_2_Sat, the ECoG and cardiovascular variables at each time point and the normalized MG counts were assessed using rank-sum test. No adjustment for multiple comparisons was undertaken at this point (17).

Spearman correlation analysis was performed and *R* values are presented where *p* < 0.05 (SPSS 19; IBM, Armonk, NY, USA). All values are expressed as means ± SEM. Statistical significance was assumed for *p* < 0.05.

## Results

### General characteristics of the experimental groups

**Figure 2:**
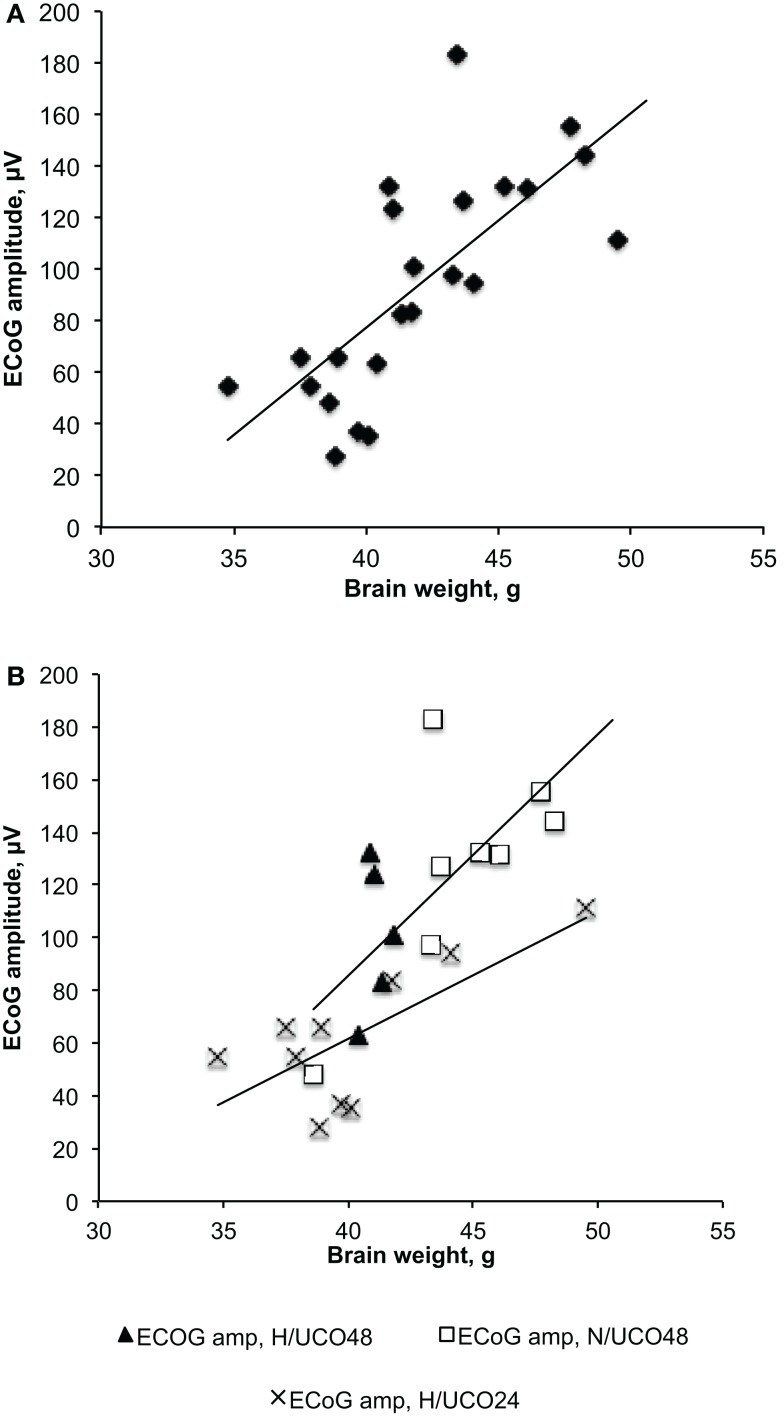
**Fetal brain weights’ correlations to the amplitudes of electrocorticogram (ECoG) at baseline, i.e., prior to commencing with umbilical cord occlusions**. **(A)** Correlation across all three groups showing increasing fetal ECoG amplitude with increasing brain weight. **(B)** Group-specific correlations still hold true despite lower brain weights in the H/UCO 24 h group compared to the N/UCO 48 h group.

Fetal acid–base status, cardiovascular parameters FHR and ABP as well as ECoG amplitudes were within physiological range in all groups except for a lower baseline ECoG amplitude in H/UCO 24 compared to the N/UCO 48 (Tables 1 and 2). In the H/UCO 24, arterial O_2_Sat measured 50 ± 3% (*p* = 0.001 versus N/UCO 48 and *p* = 0.27 versus H/UCO 48). In the N/UCO 48, arterial O_2_Sat measured 65 ± 2%, which was higher than in the H/UCO 48 where it measured 41 ± 6% (*p* < 0.05). Fetal body weights at 2.8 ± 0.2 kg in the H/UCO 24 group were lower than in both H/UCO 48 and N/UCO 48 groups at 3.7 ± 0.2 and 4.1 ± 0.2 kg, respectively (both *p* < 0.01). Fetal brain weights at 40 ± 1 g in the H/UCO 24 group were lower than in the N/UCO 48 group at 45 ± 1 g (*p* < 0.01) and not different from the values in the H/UCO 48 group at 41 ± 0.2 g. Consequently, the brain/body weight ratio at 15 ± 1 was higher in the H/UCO 24 than in N/UCO 48 group (11 ± 0.6, *p* < 0.05), but not different from that in the H/UCO 48 group at (11 ± 0.4). Across all groups, higher body and brain weights correlated with larger ECoG amplitude at baseline (*R* = 0.60 and *R* = 0.73, respectively; both *p* < 0.01). This correlation was found in the H/UCO 24 and N/UCO 48 groups with *R* = 0.73 (*p* = 0.02) and *R* = 0.70 (*p* = 0.05), respectively, but not in the H/UCO 48 group (*R* = 0.21, *p* = 0.73, Figure 2).

**Table 1:** **Acid–base status**.

	Baseline	Pattern	pH nadir
**H/UCO 24 h GROUP**			
pH	7.36 ± 0.10	7.24 ± 0.04*	6.90 ± 0.04*
Base excess (mmol/L)	3.7 ± 0.5^#^	−3.5 ± 2.1*	−16.6 ± 1.0*
Lactate (mmol/L)	1.6 ± 0.2	6.3 ± 1.5*	15.6 ± 0.3*^,#^
**H/UCO 48 h GROUP**			
pH	7.34 ± 0.01	7.23 ± 0.01*	7.01 ± 0.03*
Base excess (mmol/L)	2.0 ± 0.7	−2.8 ± 1.6*	−15.5 ± 0.3*
Lactate (mmol/L)	2.5 ± 0.9	4.8 ± 0.8*	11.9 ± 3.1*
**N/UCO 48 h GROUP**			
pH	7.35 ± 0.01	7.17 ± 0.03*	7.00 ± 0.03*
Base excess (mmol/L)	1.6 ± 0.7	−6.4 ± 1.3*	−13.6 ± 1.1*
Lactate (mmol/L)	2.0 ± 0.5	5.7 ± 1.1*	10.3 ± 1.7*

*Values are shown during baseline (prior to UCOs), between the UCOs at the onset of the synchronized ECoG–FHR pattern and at pH nadir (pH <7.00) for the hypoxic 24 h recovery group (H/UCO, *n* = 8), the normoxic 48 h recovery groups (N/UCO, *n* = 9) as well as the hypoxic 48 h recovery group (H/UCO, *n* = 5). Values are presented as mean ± SEM*.

Within group: *p < 0.05 versus baseline.

*Between groups: ^#^*p* < 0.05 for H/UCO 24 h recovery group versus N/UCO 48 h recovery group. We found no differences between the groups at the time of the synchronized ECoG–FHR pattern onset*.

**Table 2:** **Brain electrical and cardiovascular responses to umbilical cord occlusions (UCOs)**.

	Baseline	Pattern	
		dur UCO	btw UCO
**H/UCO 24 h GROUP**			
Δ*T* (min)			52 ± 13
ECoG amplitude (μV)	66 ± 12^c^	54 ± 9,c^b,c^	106 ± 21,c^a,c^
FHR (bpm)	162 ± 13	91 ± 12,b^a,b^	153 ± 12
ABP (mmHg)	42 ± 2	50 ± 3,b^a,b^	57 ± 4^a^
**H/UCO 48 h GROUP**			
ΔT (min)			59 ± 15
ECoG amplitude (μV)	100 ± 13	76 ± 10^b^	173 ± 27^a^
FHR (bpm)	168 ± 5	85 ± 7^a^	112 ± 10^c^
ABP (mmHg)	48 ± 3	60 ± 6	63 ± 6
**N/UCO 48 h GROUP**			
ΔT (min)			48 ± 12
ECoG amplitude (μV)	127 ± 14	102 ± 17^b^	209 ± 26^a^
FHR (bpm)	159 ± 5	101 ± 6,b^a,b^	171 ± 8
ABP (mmHg)	44 ± 2	57 ± 2^a^	60 ± 2^a^

*Fetal heart rate (FHR, beats per minute, bpm), arterial blood pressure (ABP), and electrocorticogram (ECoG) amplitude values are shown at baseline and during the synchronized ECoG–FHR pattern (during versus between UCOs) for the hypoxic 24 h recovery group (H/UCO, *n* = 8), the normoxic 48 h recovery groups (N/UCO, *n* = 9) as well as the hypoxic 48 h recovery group (H/UCO, *n* = 5). ΔT is calculated as the difference between the individual times of fetal adaptive brain shut-down onset (observed at the start of the synchronized ECoG/FHR change) and the time to then reach the target pH <7.00. Values are presented as mean ± SEM*.

Within group:

^a^versus baseline;

*^b^during versus between UCO (*p* < 0.01)*.

Between groups:

*^c^*p* < 0.05, H/UCO 24 h recovery or H/UCO 48 h recovery versus N/UCO 48 h recovery group*.

### Brain and cardiovascular responses to the repetitive umbilical cord occlusions

Repetitive UCOs resulted in worsening acidosis over 3–4 h with arterial pH decreasing from an average pH of 7.35 ± 0.01 at baseline to an average of 6.97 ± 0.02 across the groups and with all animals recovering to baseline by 24 h (Table 1) (12, 18). The average baseline lactate and BE were 1.9 ± 0.3 and 2.6 ± 0.4 mmol/L, respectively, increasing/decreasing to an average of 12.6 ± 1.1 and −15.1 ± 0.6 mmol/L at pH nadir, respectively (both *p* < 0.01, Table 1). Consistent with the chronic hypoxic status, in the N/UCO 48 h recovery group, baseline BE was ~1/2 that of the H/UCO 24; similarly, at pH nadir, lactate was ~2/3 that of the H/UCO 24 (both *p* < 0.05, Table 1).

Cardiovascular and ECoG responses to the UCOs have been presented elsewhere (7, 12, 18). Here, we focus on the effect of adaptive brain shut-down represented by the emergence of the ECoG–FHR synchronization as it may pertain to the neuroinflammation.

Electrocorticogram adaptive shut-down occurred 52 ± 7 min (28–69 min, 25th–75th quartiles) before reaching pH <7.00 at pH 7.23 ± 0.02 across the animal groups with no difference of Δ*T* between the groups (Table 2, Figure 1). During the adaptive shut-down, ECoG amplitude began to consistently decrease to 79 ± 9 from 164 ± 17 μV on average during versus between each UCO, respectively (*p* < 0.001). Notably, we observed no differences in pH, lactate, or BE between the groups at the time of the onset of the ECoG–FHR synchronization. The average values of arterial lactate and BE at this time were 4.8 ± 0.8 and −3.4 ± 1.6 mmol/L, respectively. These values were obtained from the most proximal blood sample taken at 20 min intervals during the experiment, i.e., usually within ~10 min from the ECoG–FHR synchronization onset.

### Effects of preceding hypoxia on cardiovascular responses and ECoG

We found no effect of chronic hypoxia on pH, lactate, or BE at any time point (baseline, emergence of ECoG–FHR synchronization or pH nadir) nor on baseline ECoG amplitude (Table 2). Moreover, chronic hypoxia preceding UCOs of increasing severity had no impact on the average timing of ~53 min prior to pH drop to <7.00 when adaptive brain shut-down was observed. There was, however, a pronounced impact of preceding hypoxia on cardiovascular and ECoG responses to the UCOs. During the ECoG–FHR synchronized activity, H/UCO 48 fetuses showed a ~35% lower mean FHR between UCOs than their normoxic counterparts (N/UCO 48) (*p* < 0.01, Table 2). During and between the UCOs, when the ECoG–FHR synchronized activity was observed, ECoG amplitude was 50% lower in the H/UCO 24 than in the N/UCO 48 (*p* < 0.05 and *p* < 0.01, respectively).

### ECoG–FHR synchronization modulates neuroinflammation and injury

**Figure 3:**
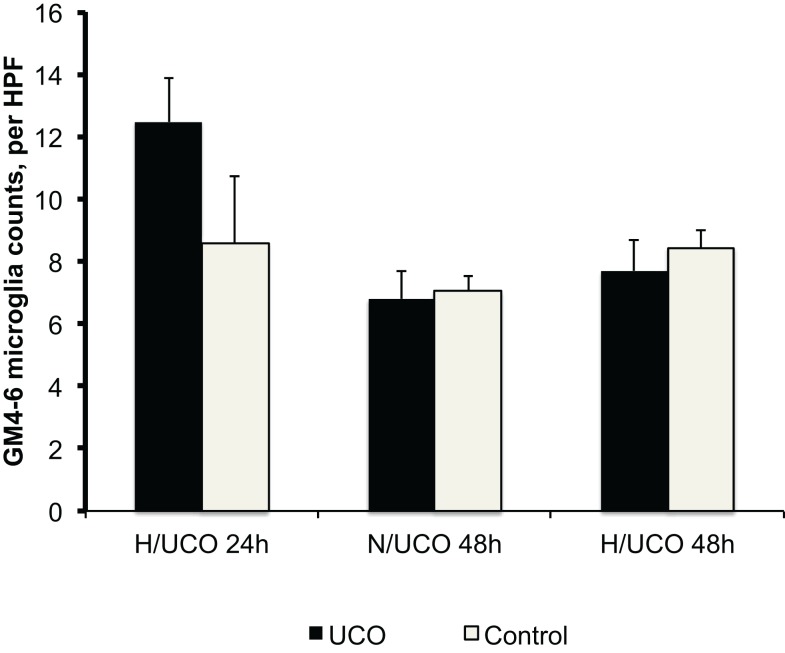
**Neuroinflammation in gray matter layers 4–6 (GM4–6) assessed as microglia (MG) counts per high-power field (HPF) at 24 and 48 h post insult in normoxic (N/UCO) and hypoxic (H/UCO) groups versus respective control groups**. Cf. Figure 3 for the inter-group comparison. H/UCO 24 data reproduced with kind permission from Elsevier.

**Figure 4:**
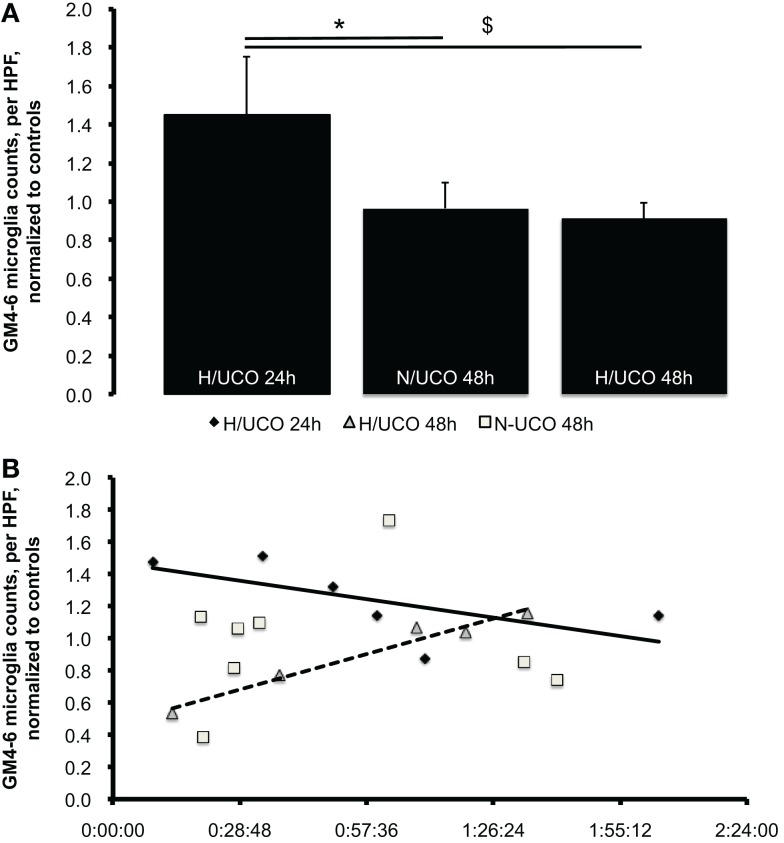
**(A)** Neuroinflammation in gray matter layers 4–6 (GM4–6) assessed as microglia (MG) counts per high-power field (HPF) at 24 and 48 h post insult, normalized by average MG counts per HPF in respective control groups’ brain regions (to allow for inter-group comparison, see “Materials and Methods” for details). Mean ± SEM. *p = 0.03 for N/UCO 48 h versus H/UCO 24 h group; ^$^*p* = 0.02 for H/UCO 48 h versus H/UCO 24 h group. **(B)** Correlation to adaptive brain shut-down timing expressed by Δ*T* as the difference between the individual times of fetal adaptive brain shut-down onset (observed at the start of the synchronized ECoG/FHR change) and the time to then reach the target pH <7.00. Spearman correlation coefficients *R* = −0.84 for H/UCO 24 h group and *R* = 0.90 for H/UCO 48 h group (both *p* = 0.04); no significant correlation for N/UCO 48 h group (*R* = −0.05, *p* = 0.91). Due to artifacts in ECoG, Δ*T* was missing in two out of eight H/UCO 24 h group animals and in one animal from the N/UCO 48 h group.

To test the hypothesis that neuroinflammation is diminished by adaptive brain shut-down, we studied MG counts in the GM4–6, the brain region where most ECoG activity is generated (Figure 3). Within each group, there were no differences between the MG counts in the GM4–6 region of UCO group animals compared to the respective controls. Normalized GM4–6 MG counts were higher in H/UCO 24 animals than H/UCO 48 and N/UCO 48 animals (both *p* < 0.05; Figure 4). In contrast, MG counts in the N/UCO 48 animals were not different from those in the H/UCO 48 animals. Supporting our hypothesis, MG counts were inversely correlated to Δ*T* in the H/UCO 24 animals (*R* = −0.84), but not in N/UCO 48 animals, and surprisingly in H/UCO 48 animals this relationship was reversed (*R* = 0.90, Figure 4).

## Discussion

The chief findings of this study are that higher MG counts in GM4–6 of the H/UCO 24 compared to both H/UCO 48 and N/UCO 48 animals indicate a rapid onset/offset of the fetal brain inflammatory response with repetitive UCOs and severe acidemia, while the inverse correlation to ΔT supports the earlier onset of ECoG shut-down as an important neuroprotective mechanism limiting MG activation at 24 h post UCO insult. In contrast, albeit at a lower level compared to the H/UCO 24, we found relatively higher MG counts in the H/UCO 48 in those fetuses that showed an earlier adaptive brain shut-down. How can we explain this time-specific effect of the shut-down of neuronal metabolism on neuroinflammation?

### Does fetal chronic hypoxia alter cerebral response to an acute hypoxic-acidemia insult?

It is possible that chronic hypoxia primes fetal brain re-exposure to an inflammatory stimulus such as acidemia, which then is potentiated or induced by (further) lactate accumulation during an adaptive brain shut-down triggered by worsening acidemia. This may be due to several inter-related reasons. First, fetal brains may be less capable of metabolizing the lactate primarily due to pre-existing hypoxia with curbed metabolism and lesser ATP reserves to sustain the anti-inflammatory adenosine mono-phosphate kinase (AMPK) activation status beyond an acute response (19–22). Second, altered cardiovascular responses to UCOs in the H/UCO group fetuses with resulting stress on the auto-regulatory capacity of the cerebral blood flow may further contribute to the gradual exhaustion of the cerebral metabolic reserves in these fetuses (23, 24). Third, hypoxic fetal brains that shut-down earlier may accumulate at 48 h post UCOs a relatively higher level of regional lactate and other inflammatory mediators.

Rising cerebral extracellular acidosis reduces EEG seizure activity via activation of inhibitory interneurons by acid-sensing ion channels 1a (ASIC1a) (25). Thus, even extracellular brain acidosis *without* hypoxia increases neuronal inhibitory tone. This lends support to our hypothesis that the observed cyclical changes in amplitude and frequency of the fetal ECoG during the ECoG–FHR synchronization at arterial pH ~7.23 and lower are due to unmasking of inhibitory interneuron activity during the UCOs (7). Metabolic, respiratory, or mixed acidemia with pH <7.25, also in absence of hypoxia, results in reversible EEG changes in preterm neonates of <32 weeks with increasing inter-burst intervals indicating a global alteration of cerebral blood flow and related shut-down of brain electrical activity (26). Other mechanisms may also be involved in the observed ECoG dynamics and ensuing neuroinflammation. They may include depolarization block and adenosine (A1) receptor-mediated decrease in neuronal activity. (27, 28) It remains to be explored whether these processes can explain the temporal profile of the ECoG changes we have observed.

The requirement of acidemia to produce neuroinflammation is supported by the findings in fetal sheep near term that chronic hypoxia does not lead to microglial activation and acidemia is required to induce neuroinflammation (9, 29). Moreover, isolated systemic hypoxia without ischemia does not cause neuronal necrosis as shown in middle cerebral artery occlusion model of stroke in adult rats, however, it will exacerbate ischemic necrosis (30). In line with these findings, pediatric patients <9 months old with proven hypoxic or hypotensive episodes due to perinatal asphyxia, congenital heart defects, or chronic pulmonary dysfunction were found to often show a dense infiltrate of microglial cells in the dendate gyrus making this neuroinflammation a neuropathological marker of mild hypoxic–ischemic brain injury (31). That is, neuroinflammation, preceding brain necrosis, plays a pivotal role in mediating brain injury induced by neonatal hypoxic–ischemic encephalopathy, the neuropathological substrate of cerebral palsy (32). Meanwhile, suspending neuronal activity is known to result in prolonged preservation of viability in adults and neonates (33, 34). This makes our present observations plausible that neuroinflammation is mitigated by an early adaptive neuronal shut-down in response to repetitive intermittent hypoxic episodes with cumulative acidemia.

### Methodological considerations

As outlined in Section “Materials and Methods,” we believe that the difference in experimental design had no confounding impact on our findings, since both approaches produced a worsening acidemia within a similar time frame. This is also supported by the fact that all groups had normal pH values at baseline and that the adaptive brain shut-down was uniformly observed around the same time and around the same pH, lactate, and BE values. Moreover, all groups recovered their blood gas and acid–base status to baseline within 24 h as reported (7, 12, 18). However, the chronic hypoxia in the animals of the H/UCO 24 was accompanied by a higher BE at baseline and reaching a higher lactate at pH nadir than in the N/UCO 48. Moreover, the correlation between the timing of onset of adaptive brain shut-down and neuroinflammation was not observed in the N/UCO 48, but only in both chronically hypoxic UCO groups. Thus, we propose that any impact of the experiments on the neuroinflammation measured at 24 or 48 h of recovery is due to the presence or absence of preceding spontaneous chronic hypoxia. The ECoG amplitude difference between the H/UCO 24 and N/UCO 48 groups is likely attributable to differences in the animal size as we report herein. While the expected relationship between brain weight and ECoG amplitude held true in both groups, the generally lower brain weight in the H/UCO 24 group explains the lower ECoG amplitude in this group compared to N/UCO 48 group. This finding is further supported by the evidence of the asymmetric IUGR in the H/UCO 24 group, at least in comparison to the N/UCO 48 group. IUGR, especially an asymmetric IUGR, has been associated with impaired synaptogenesis and altered ECoG activity (35–37). Lower ECoG amplitude in conjunction with the above indicators of altered growth trajectory supports the notion that the H/UCO 24 group was different from N/UCO 48 group in the intended sense of being chronically hypoxic.

A weakness of the present study is that we could not evaluate the effect of UCOs on neuroinflammation at 24 h in fetuses that were normoxic to match the findings at 48 h in the N/UCO 48. Consequently, our data currently suggest that earliness of the onset of the adaptive brain shut-down confers no neuroprotection in the sense of reduced neuroinflammation to the fetuses that were normoxic prior to UCOs. Next, while adaptive brain shut-down represents a global brain response, most ECoG activity is generated in the cortical gray matter layers 4–6. Hence, here we reasoned that our primary focus in connecting ECoG to neuroinflammation should be on the microglial activity in these cortical layers. However, upper cortical layers and subcortical afferents also contribute to ECoG, albeit likely on a subtler level of ECoG properties that is not captured by the visually discernable ECoG changes characteristic of the adaptive brain shut-down. Hence, further studies are needed to delineate this relationship further and under varying conditions leading up to an adaptive brain shut-down.

### Conclusion and significance

The translational significance of our findings is that EEG–FHR monitoring during labor may not only identify fetuses at risk of developing severe acidemia ~1 h ahead of the pH drop to <7.00; EEG–FHR monitoring intrapartum may also provide information to the neonatologist as to which babies may be at higher risk of brain injury resulting in conditions such as cerebral palsy due to developing neuroinflammation. FHR monitoring may guide this diagnostic process by helping to distinguish chronically hypoxic fetuses prior to labor onset from the normoxic fetuses. The therapeutic implication is that neuroprotective drug treatments need to target the postnatal 24 h time window to benefit from the anti-neuroinflammatory effect the adaptive brain shut-down may have in the chronically hypoxic fetuses when they are born.

## Conflict of Interest Statement

Bryan S. Richardson and Martin G. Frasch are inventors of related patent applications entitled “EEG Monitor of Fetal Health” including US Patent Application Serial No. 12/532,874 and CA 2681926 National Stage Entries of PCT/CA08/00580 filed March 28, 2008, with priority to US provisional patent application 60/908,587, filed March 28, 2007. No other disclosures have been made.

## Presentation Information

Presented in part at Society for Gynecologic Investigation Annual Meeting 2013.
